# Medicinal and economic benefits of legalization of marijuana in Pakistan

**DOI:** 10.17179/excli2022-5477

**Published:** 2022-11-02

**Authors:** Anum Khalid, Abdul Haseeb, Gohar Mushtaq, Mohammad A. Kamal

**Affiliations:** 1 Faculty of Medicine, Karachi Medical and Dental University, Karachi, Pakistan; 2 Center for Scientific Research, Faculty of Medicine, Idlib University, Idlib, Syria; 3 Institutes for Systems Genetics, Frontiers Science Center for Disease-Related Molecular Network, West China Hospital, Sichuan University, China; 4 King Fahd Medical Research Center, King Abdulaziz University, Saudi Arabia; 5 Department of Pharmacy, Daffodil International University, Bangladesh; 6 Enzymoics, NSW 2770; Novel Global Community Educational Foundation, Australia

## ⁯⁯

Cannabis (marijuana) belongs to a class of drugs that has been increasingly utilized in the last few decades (Hajizadeh, 2016[5]). The drug is derived from the plant Cannabis sativa (hemp), and it interacts with the endocannabinoid system via type 1 (CB1) and type 2 (CB2) receptors in the body. Type 1 receptors are concentrated in the nervous tissues, with smaller distributions in the smooth muscle, myocardium, and adipocytes. Conversely, type 2 receptors are more prevalent in peripheral blood mononuclear cells, including macrophages, B cells, and Natural Killer cells (Ebbert et al., 2018[3]).

Cannabis has been used for medicinal purposes to treat patients with various indications suitable for cannabis therapy such as chronic pain and muscle spasticity (Hajizadeh, 2016[5]). The drug has been proven effective in treating patients who experience severe nausea due to chemotherapy or other medicinal treatments (Page et al., 2020[9]; Dhadwal and Kirchhof, 2018[2]; Elliott et al., 2016[4]). Studies have demonstrated the potential of cannabis as a drug that facilitates wound healing and reduces melanoma symptoms (Dhadwal and Kirchhof, 2018[2]). However, the debate surrounding its medicinal benefits and adverse effects is still ongoing (Hammond et al., 2020[7]).

Several adverse effects have been associated with the use of cannabis. It has the potential to intoxicate the user, which may lead to irrational activities such as road accidents (Hall, 2020[6]). When cannabis users are not intoxicated, they may experience other symptoms such as cognitive impairments and mood disorders. When used in the inhaled form, cannabis smoke exerts similar deleterious effects on the lungs as those of tobacco smoke such as chronic bronchitis, increased sputum with cough production and hyperinflation (Yayan and Rasche, 2016[10]). Prolonged use of the drug can induce psychotic disorders (Bridgeman and Abazia, 2017[1]).

It is illegal to sell or purchase cannabis in Pakistan, which increases the pressure on law enforcement authorities to regulate the use and abuse of this drug. Cannabis being an illicit drug pushes people towards criminal offenses such as selling or purchasing the drug for personal use or otherwise (Dhadwal and Kirchhof, 2018[2]). Statistical data illustrate that in 2018, in Karachi city alone 41.96 metric tons of cannabis were consumed (McCarthy, 2019[8]). The government, in turn, has to invest additional resources to implement the strict policies and punishments for such offenses and offenders, respectively. It is difficult to regulate the use of the drug because of its current illegal status. Another problem associated with the strict legal policies is the difficulty of procurement of cannabis for research and medical use. 

Legalizing cannabis in Pakistan would be a landmark change in policy, as it will help to regulate the drug. There will be reduced incidences of criminal offenses, and the drug will be dispensed and purchased under effective regulatory policies. The government can introduce tax on the drug, which adds to the economy (Dhadwal and Kirchhof, 2018[2]). Physicians in Pakistan can prescribe medicinal cannabis to treat patients with chronic conditions, thus improving the quality of life of those patients. If the legal status of cannabis use is approved in Pakistan, a nationwide patient registry can be created to provide comprehensive data regarding the conditions for which patients use cannabis to better understand the trends in the use of cannabis and its potential effectiveness. Finally, easy procurement of the drug can allow the researchers to conduct observational and well-controlled clinical trial studies on the safety and efficacy of medicinal cannabis therapies, which can pave the way for future discoveries.

## Conflict of interest

The authors declare no conflict of interest.
